# The effect of acupuncture on stroke recovery: study protocol for a randomized controlled trial

**DOI:** 10.1186/1472-6882-12-216

**Published:** 2012-11-12

**Authors:** Huilin Liu, Dangsheng Zhang, Xiuge Tan, Daqing Yang, Guiling Wang, Yin Zhao, Yali Wen, Guangxia Shi, Linpeng Wang

**Affiliations:** 1 Acupuncture and Moxibustion Department, Beijing Traditional Chinese Medicine Hospital affiliated with Capital Medical University, 23 Mei Shu Guan Hou Street, Beijing, 100010, China; 2 Neurology Department of Beijing Shunyi District Hospital, Beijing, China; 3 Neurology Department of Beijing Pinggu District Hospital, Beijing, China; 4 Traditional Chinese Medicine Department of Liangxiang Hospital of Beijing Fangshan District, Beijing, China

## Abstract

**Background:**

Stroke is one of the leading causes of death and disability in China. Current treatments for stroke are limited and achieve no optimal effect. Acupuncture is widely used in the treatment of stroke and in improving the quality of life for patients in China. In most previous clinical studies, the effects of acupuncture have been diverse, and few well-designed randomized controlled trials have been conducted to investigate the long-term effect of acupuncture on acute stroke recovery.

**Method:**

Three hundred and twenty eight subjects with acute cerebral apoplexy will be recruited. The patients will be randomized into two different groups: the intervention group will receive acupuncture treatment together with Western standard treatment for 2 weeks plus the secondary prevention treatment for 22 weeks; the control group will receive only the Western standard treatment for 2 weeks and the secondary prevention treatment for 22 weeks. The primary outcome measures are Barthel Index and the Stroke-Specific Quality Of Life. The secondary outcome measures are the National Institutes of Health Stroke Scale and Modified Rankin Scale. All assessments will be conducted at the baseline and at weeks 4, 12 and 24 of follow-up.

**Discussion:**

This study will evaluate the effects of acupuncture on the long-term recovery of acute stroke and on improving the quality of life of the patients. The results of this study will help establish optimal integrated therapeutic strategies for patients with stroke.

**Trial registration:**

Current Controlled Trials ISRCTN29932220

## Background

With the rapid economic development in China, the Chinese healthcare system has seen significant improvements. However, tough challenges remain in the treatment of stroke, the leading cause of death and disability in China [1]. Although stroke is a global epidemic, about 85% of all stroke deaths are registered in low- and middle-income countries, and patients in these countries also account for 87% of total stroke-related losses in terms of disability-adjusted life years [2]. For example, in China, stroke morbidity is 89.6–314 out of every 100,000 males and 76.7–212.2 out of every 100,000 females. The mortality from stroke in urban areas is 127.96 out of every 100,000 people, and in rural areas 115.2 out of every 100,000 people [3].

Poor control of vascular risk factors and limited access to acute and primary care are the most common reasons for the high mortality and rate of disability after stroke. In addition, secondary prevention-an important possible approach in the treatment of stroke-is also largely lacking in low- and middle-income countries [4].

Despite the availability of effective therapies, optimal management of stroke remains problematic. Current therapeutic strategies focus on restoring blood flow in ischemic strokes, and on controlling bleeding in hemorrhagic strokes. These approaches are not effective for long-term stroke recovery or for enhancing the quality of life of patients. For example, thrombolytic therapy carries the risk of hemorrhage and is limited to the first three hours after the stroke [5-7].

Acupuncture has been practiced in China for more than 3,000 years [8], and is traditionally considered as a treatment for various ailments associated with stroke. Recently, there have been many reports of its benefits in the treatment of stroke [9-11]. However, the effect size of these clinical studies was underestimated because of methodological flaws, including inadequate statistical analysis, a failure to describe differences in baseline stroke severity, and an inadequate assessment period [11,12]. Thus, acupuncture may be more effective in assisting stroke recovery than has generally been reported, especially when the stroke is moderately severe.

In this study protocol, we describe the design of a parallel multi-center randomized controlled trial. The aims of the study are to provide an alternative strategy for stroke treatment and thereby improve stroke care, to illustrate the potential benefits of acupuncture in stroke recovery, and to enhance the life quality of patients with acute ischemic stroke.

## Methods/design

### Study design

**Figure 1:**
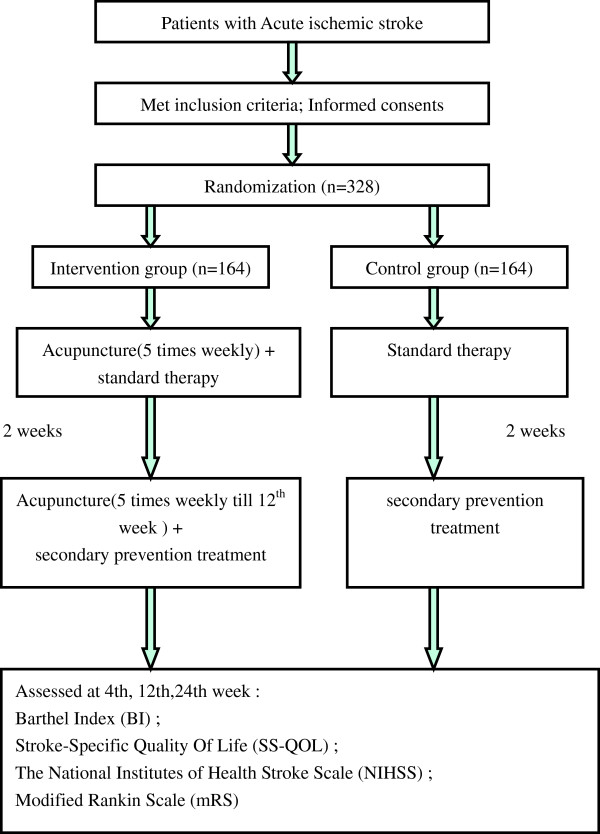
Flowchart of the study design.

The study is a parallel multi-center randomized controlled trial. It will compare the combination of acupuncture and Western medicine treatments with standard Western stroke treatment given alone during the acute phase (24 weeks) after stroke. The Barthel Index (BI) and Stroke-Specific Quality Of Life (SS-QOL) will be assessed as primary outcome measures, and the secondary outcome measures are the National Institutes of Health Stroke Scale (NIHSS) and modified Rankin Scale (mRS). All assessments will be conducted at baseline and at weeks 4, 12 and 24 of follow-up. The study design is detailed in Figure 1.

### Ethics

The study is being conducted in accordance with the Medical Research Council Ethics Guidelines. Ethical approval and scientific review of this study were obtained from the Research Ethical Committee of the Beijing Hospital of Traditional Chinese Medicine on 22 January 2010 (Ref: 201002–1).

### Informed consent

Prior to the study, participating patients will receive written and oral information about the study process and the required time commitment.

### Patients

All patients admitted to the Neurology Department of Beijing Shunyi District Hospital and Beijing Pinggu District Hospital with acute ischemic stroke will be considered for recruitment. Patients of any age with a recent (within 14 days), clinically and computer tomography/magnetic resonance imaging-confirmed ischemic stroke who are able to give informed consent will be eligible for inclusion.

#### Inclusion criteria

The inclusion criteria are: (1) stroke diagnosed according to the criteria of cerebral arterial thrombosis in Western medicine and apoplexy in Chinese medicine; (2) acute cerebral arterial thrombosis that occurred in the past 14 days; (3) male or female aged 40–75 years; (4) NIHSS grade from 4 to 21; (5) Glasgow Coma Score ≥ 7; (6) patients with their first attack or with a history of cerebral stroke but with no serious deformity and mRS grade ≤ 1; and (7) voluntary participation in the trial and provision of informed consent.

#### Exclusion criteria

The exclusion criteria are: (1) ongoing thrombolytic treatment; (2) participation in other clinical trials, currently or in the last 3 months; (3) severe primary diseases of the cardiovascular system, liver, kidney, or hematopoietic system; (4) psychological disorders; (5) pregnancy or breast-feeding; and (6) inborn handicaps.

### Randomization

The computer-generated randomization is conducted by the Epidemiological Research Center of the Third Affiliated Hospital of Beijing University. Sequential, sealed, opaque envelopes are used for allocation, which occurs after the acupuncturist’s evaluation (concealed allocation).

### Interventions

A total of 328 patients in the acute stage of cerebral apoplexy will be recruited. The patients are randomly divided into two different groups: 1) the intervention group and 2) the control group. The intervention group will receive acupuncture treatment in addition to the Western medicine standard treatment for 2 weeks and the secondary prevention treatment for 22 weeks, and the control group will receive standard Western-medicine therapy for 2 weeks and the secondary prevention treatment for 22 weeks. Both groups will be evaluated at baseline and at 4, 12 and 24 weeks of treatment.

#### Intervention group

Seven main acupoints will be stimulated: Baihui (Du-20), Quchi (LI-11), Shousanli (LI-10), Hegu (LI-4), Zusanli (ST-36), Yanglingquan (GB-34), and Sanyinjiao (SP-6). Acupoints association will be used according to the theory of Chinese medicine and the unique symptoms of the patients. The following additional acupoints are commonly used: Jianyu (LI-15), Huantiao (GB-10), Qiuxu (GB40), twelve well-jing points, Jinjin (EX-HN 12), Yuye (EX-HN 13), Yamen (DU-15), Lianquan (RN 23), Tianshu (ST-25), Fenglong (ST-40), and limb spasm assists with fire needles.

The acupoints will be stimulated by filiform stainless-steel needles (0.30 mm diameter, 40 mm length). Patients in the acute stroke stage will be manually stimulated to elicit needle sensation (de qi) at the main acupoints detailed above, with a needling depth of 30 mm. The needles will remain for 30 minutes per treatment, and the treatments are given five times per week. At the same time, Jinjin (EX-HN 12), Yuye (EX-HN 13), Sishencong (EX-HN 1) and the twelve jing well points will be stimulated by quick insertion of the tri-ensiform needles to induce bleeding. The stimulation will last for two weeks. Stimulation of Jianyu (LI-15), Huantiao (GB-10), Qiuxu (GB40), Yamen (DU-15), Lianquan (RN 23), Tianshu (ST-25) and Fenglong (ST-40) will be performed according to the specific syndrome of each patient. For instance, Jianyu (LI-15) and Huantiao (GB-10) will be selected for hemiplegic paralysis; Yamen (DU-15) and Lianquan (RN 23) for aphasia; and Tianshu (ST-25) and Fenglong (ST-40) for constipation.

Western medicine standard therapy, such as inhibition of platelet aggregation, control of blood pressure, routine physiotherapy, occupational therapy, glucose control, dyslipidemia control, and dehydration treatment if necessary, will be given for 2 weeks. The secondary prevention treatments, such as blood pressure control, inhibition of platelet aggregation, glucose control, and dyslipidemia control, will be given for 22 weeks.

#### Control group

The patients will receive the standard therapy for 2 weeks and the secondary prevention treatment for 22 weeks as mentioned above.

### Outcome measures

#### Participant demographics and general status

Demographic information such as sex, age, marital status, educational background, indicators of social economic status (e.g. household income) and time from attack will be extracted from baseline questionnaires. Vital signs (respiration rate, pulse, blood pressure and temperature) will be measured by nurses.

#### Primary

##### The BI

The BI is a scale that measures ten basic aspects of activity related to self-care and mobility [13]. For the Chinese version, the BI is translated into Chinese, and the ten items are: feeding, bathing, grooming, dressing, continence of bowels and bladder, transferring to and from a toilet, moving from wheelchair to bed and return, walking on level surface for 45 meters, and going up and down stairs. The sum of the scores for the individual items forms the total score. The normal score is 100, and lower scores indicate greater dependency. If a person manages about 50% of the items independently, their score would fall in middle.

The BI will be assessed by the investigators at baseline and at 4, 12 and 24 weeks after the stroke. The total BI scores will be recorded and classified into three groups: independent (BI score ≥ 60), with help (BI score 40–60), and dependent (≤ 40).

##### The SS-QOL

The SS-QOL is a patient-reported outcome measure intended to provide an assessment of health-related quality of life, specific to patients with stroke [14]. The SS-QOL questionnaire consists of 49 items in the 12 domains of energy, family roles, language, mobility, mood, personality, self-care, social roles, thinking, upper extremity function, vision, and work/productivity. Scoring of the SS-QOL is rated on a 5-point Likert scale. Response options are scored as 5 (“no help needed/no trouble at all/strongly disagree”), 4 (“a little help/a little trouble/moderately disagree”), 3 (“some help/some trouble/neither agree nor disagree”), 2 (“a lot of help/a lot of trouble/moderately agree”), and 1 (“total help/could not do it at all/strongly agree”). The domains are scored separately, and a total score is also calculated, with higher scores indicating better function.

An SS-QOL version translated to Chinese will be provided to the patients at baseline and at 4, 12 and 24 weeks. The test-retest reliability, internal consistency, construct, and convergent validity of the SS-QOL in patients with stroke will be ascertained by the investigators.

#### Secondary

##### The NIHSS

The NIHSS is a standardized stroke severity scale used to describe neurological deficits in stroke patients. The NIHSS comprises tests of 11 items, including the level of consciousness, selected cranial nerves, motor function, sensory function, cerebellar function, language, and inattention (neglect) et al. Total scores range from 0 to 42, with scores above 25 indicating very severe neurological impairment, scores of 5–24 suggesting moderately severe to severe impairment, and scores below 5 indicating mild impairment [15].

Each patient will be scored by a trained, certified investigator at the time of initial stroke evaluation (baseline) and at 4, 12 and 24 weeks after treatment.

##### The mRS

The mRS is another commonly used scale that measures disability or dependence in activities of daily living in stroke victims [16]. Consenting patients with stroke in this trial will have the mRS performed by the investigators at baseline and at 4, 12 and 24 weeks.

#### Follow-up

Assessments will be conducted at baseline and at weeks 4, 12 and 24 of follow-up. The follow-up assessment is designed to evaluate the long-term effect of acupuncture treatment.

### Quality control

The staff will be specially trained to treat stroke patients; they will take part in special education programs to improve their knowledge of stroke. They will be trained to know the inclusion and exclusion criteria, and all the occupational therapists will master the acupuncture methods used in this trial.

Patients will be allocated to different groups using a random number table to avoid selection bias. Blinding will be used both for the evaluators and the patients to avoid measurement bias and the influence of psychological factors on the therapy effect. All statistical analysis will be conducted by the statistician, who will be blinded to the grouping and analysis procedure.

### Statistical analysis

The statistical analysis will be performed by the Epidemiological Research Center of the Third Affiliated Hospital of Beijing University.

The quantitative data are presented as mean ± standard deviation and will be analyzed using analysis of variance when normally distributed. Non-parametric data will be analyzed using the Wilcoxon test with repeated measures. Enumeration data are presented as frequencies or ratios. If the outcome variables are two-way 2×2 contingency tables, chi-square tests or Fisher exact probability tests will be performed. If they are one-way 2×4 ordered data, Ridit analyses will be performed.

Statistical analyses will be conducted using SPSS version 13.0 (SPSS, Chicago, IL, USA). The P-value cut-off for statistical significance is defined as *P* < 0.05, and all statistical tests are two-tailed.

## Discussion

Acupuncture is widely used to enhance stroke recovery in Eastern countries. However, a recent randomized controlled trial demonstrated that acupuncture has no specific efficacy in stroke rehabilitation [17]. A meta-analysis of sham-controlled randomized clinical trials concluded that there is insufficient evidence to support the use of acupuncture for functional recovery after stroke [18]. These data have led to ongoing controversy regarding the use of acupuncture therapy after stroke, despite the thousands years of evidence from traditional Chinese medicine practice.

The occupational therapists’ technique in the manipulation and selection of acupoints is the most important factor that determines the efficacy of acupuncture therapy [19]. In the present study, all occupational therapists are experts with more than ten years of acupuncture practice and plenty of experience with stroke treatments. The acupoints selected in this trial correspond to a complex prescription for stroke therapy originating in over 60 years of clinical application. Moreover, all the occupational therapists will receive special training to achieve a sound understanding of the acupoint selection and to normalize the practices across different therapists. The trial complies with the STRICTA (STandards for Reporting Interventions in Controlled Trials of Acupuncture).

The efficacy of acupuncture is different at different levels of stroke severity. It may have limit efficacy in very severe strokes, particularly in cases of extensive brain damage [20,21]. However, for mild or moderately severe strokes, acupuncture might be a superior treatment. In this trial, we will recruit patients with moderately severe acute strokes without severe deformity, and with NIHSS grades from 4 to 21, Glasgow Coma Scores of greater than or equal to 7, and mRS grades of less than or equal to 1.

Recovery of function after stroke is a long steady process that continues for several weeks or even months before reaching a stable phase. The effects of acupuncture occur within the same time frame. Several previous studies did not show any beneficial effect of acupuncture, and this may in part be explained by an assessment of efficacy too soon after acupuncture treatment (in most cases 2–3 weeks after stroke) [22,23]. An assessment or reassessment at a minimum of 4 weeks, and ideally at least 3 to 6 months after the completion of treatment is essential. We followed the time course of the normal process of stroke recovery in designing our study protocol. Thus, we will assess efficacy at baseline and at 4, 12 and 24 weeks after acupuncture intervention.

The primary endpoint for this study is the change in the quality of life in the intervention and control groups, from baseline to 4, 12 and 24 weeks post-therapy. Neurological deficits, disability, and dependence in activities of daily living will be evaluated by NIHSS and mRS as the secondary endpoints.

Many acupuncturists consider the main effect of acupuncture on the human body after stroke to be the rehabilitation of motor function, whereas few have considered the patients’ life quality, which includes factors such as dependence in activities of daily living, feeding, dressing, and continence of bowels and bladder. To address this, we will use the BI and SS-QOL questionnaires to measure the quality of life as the primary outcomes.

## Competing interests

The authors declare that they have no competing interests.

## Authors’ contributions

Liu HL wrote and revised the manuscript and obtained research funding, Wang LP conceived the study, Yang DQ and Shi GX wrote the first draft of the paper. Zhang DS and Tan XG will oversee delivery of the intervention, Wang GL and Zhao Y will manage the trial and collect the data, and Wen YL will undertake data analyses. All authors contributed to drafting the paper and all authors have read and approved of the final version of the manuscript.

## Pre-publication history

The pre-publication history for this paper can be accessed here:

http://www.biomedcentral.com/1472-6882/12/216/prepub
